# Perspective: Soybeans Can Help Address the Caloric and Protein Needs of a Growing Global Population

**DOI:** 10.3389/fnut.2022.909464

**Published:** 2022-05-06

**Authors:** Mark Messina

**Affiliations:** Soy Nutrition Institute Global, Washington, DC, United States

**Keywords:** soybeans, protein, versatility, efficiency, sustainable, requirements, soyfoods

## Abstract

Feeding a growing global population with projected rising socioeconomic status will require additional sources of calories and especially protein. These sources need to align with the Sustainable Development Goals established by the Food and Agriculture Organization of the United Nations. The soybean is uniquely positioned to meet this challenge based on the following criteria: (1) Global soybean production exceeds by ~4 times the production of all pulses combined (2) Soybeans are higher in protein than other legumes and soy protein quality is similar to animal protein quality (3) Soybeans are an excellent source of healthy fat, including both essential fatty acids (4) Soybeans, like other legumes, symbiotically fix atmospheric nitrogen thereby reducing the need for fertilizer inputs (5) Greenhouse gas emissions per unit protein are lower than for nearly all other foods (6) Soybeans, like other legumes, are also recognized as an affordable food that can be incorporated into diverse diets regardless of economic standing and (7) The range of foods produced from soybeans constitutes an important position in historic and contemporary cuisines, cultures and emerging consumer trends for plant-based protein. Although most soybeans are currently used for animal feed, soybean use is dictated by consumer demand. Therefore, soybeans are well positioned to meet future global needs for energy and protein. Armed with this knowledge, health professionals can feel justified in encouraging greater consumption of soyfoods for both personal and planetary reasons.

## Introduction

Sustainably producing sufficient food, and especially protein, for a global population expected to reach 9.7 billion by 2050 represents a significant challenge (1). Sustainable food patterns are those that are nutritionally adequate, economically affordable, socially acceptable, and conserve both agroecosystems and biodiversity (2). Consumers (3) and health and nutrition organizations (4, 5), including the Food and Agriculture Organization (FAO) of the United Nations (6), recognize the importance of meeting this challenge. In our view, the soybean (*Glycine max* (L.) Merr.) is uniquely positioned to help meet the caloric and protein needs of the growing global population.

The protein content of the soybean is higher (Table 1) than other legumes as is the quality of its protein (7). In fact, soybeans produce the highest protein yield per hectare (8) and are produced at a global scale far exceeding other legumes and pulses. For example, for the year 2020, ~13-fold more soybeans were produced than were produced of the common bean (~353 vs. ~28 million metric tons [MT]), which is the bean produced in the highest quantity aside from soybeans. These attributes are notable because evidence suggests that (1) the US protein recommended dietary allowance [RDA: 4–13 y, 0.95 g/kg; 14–18 y, 0.85 g/kg; ≥19 y, 0.80 g/kg (9)] may be too low for optimal health (10, 11) and (2) if supply and economic conditions allow, populations will elect to consume more protein than the RDA. Additionally, because of its high fat content (~20% of the dry weight), the caloric density of the soybean exceeds that of other beans, so it can more readily contribute to meeting energy requirements and the requirements for both essential fatty acids, the omega-6 fatty acid linoleic acid and the omega-3 fatty acid alpha-linolenic acid, which represent approximately 54.4 and 7.9% of the total fat content, respectively (12).

**Table 1:** Macronutrient, caloric and fiber content per 100 g of legumes (boiled unless otherwise indicated) according to the USDA nutrient database.

**Legume**	**USDA** **FDC ID**	**kcal**	**Protein**		**Fat**		**Carbohydrate**		**Fiber**	**Density**
			**g**	**% kcal**	**g**	**% kcal**	**g**	**% kcal**	**G**	**Kcal/g**
Lupin	173804	116	15.57	53.69	2.92	22.7	9.29	32.0	2.8	1.16
Soybeans	174271	172	18.21	42.35	8.97	46.9	8.36	19.4	6.0	1.72
Lentils	175254	114	9.02	31.65	0.38	3.0	19.54	68.6	7.9	1.14
Pinto beans	175200	143	9.01	25.20	0.65	4.1	26.22	73.3	9.0	1.43
Great northern	173790	118	8.33	28.24	0.45	3.4	21.09	71.5	7.0	1.18
Kidney beans (red)	175242	127	8.67	27.31	0.5	3.5	22.8	71.8	7.4	1.27
Black beans	175237	132	8.86	26.85	0.54	3.7	23.71	71.8	8.7	1.32
Mung beans	175255	125	7.02	26.74	0.38	3.3	19.15	73.0	7.6	1.25
Peas (green)	170102	84	5.36	25.52	0.22	2.4	15.63	74.4	5.5	0.84
Navy beans	173794	140	8.23	23.51	0.62	4.0	26.05	74.4	10.5	1.40
Adzuki beans	173789	128	7.52	23.50	0.1	0.7	24.77	77.4	7.3	1.28
Lima beans	169316	123	6.81	22.15	0.32	2.3	23.64	76.9	5.3	1.23
Garbanzo beans	173799	164	8.86	21.61	2.59	14.2	27.42	66.9	7.6	1.64
Peanuts (raw)	172432	570	25.09	17.61	47.58	75.1	20.91	14.7	8.7	5.70

Furthermore, the wide range of foods that can be produced from soybeans, from the traditional Asian fermented (miso, natto, tempeh) and unfermented (tofu, soymilk, edamame, soynuts) soyfoods to modern soyfoods that use soy protein as a base to create energy bars, drinks, and meat and dairy alternatives, means the soybean can be embraced by populations with diverse cuisines. Importantly, the acreage devoted to soybean production dwarfs that of other dry beans (8) and growing soybeans has been shown to be a highly energy efficient and sustainable way to produce protein (13). Therefore, the scale, supply, and production systems for soybeans meet the challenge at hand. Finally, legumes in general are considered to be an affordable source of protein, thereby providing a nutrient dense solution for consumers under a range of socioeconomic conditions (14) Table 1.

## Dietary Protein Requirements

### Population Requirements

Valin et al. (15) estimated that relative to 2005 levels, global food demand would grow by 50 to 100% by 2050 due to an increase in per capita consumption. Protein demand will also substantially increase (16) due to population growth and the positive relationship between increasing incomes and protein intake (especially animal protein) (17). In 2017, Henchion et al. (18) projected that if each person consumes current maximum protein consumption levels (estimated at 103 g/d), protein production would need to increase by 78% to meet the needs of an expected population of 9.6 billion by 2050. Consistent with this estimate, Lieberman et al. (19) found that among 14 developed countries, protein intake was consistently ~16% of total energy, which is nearly twice the percentage of calories derived from protein needed to meet the adult RDA (10). This finding is consistent with the protein leverage hypothesis (20), which maintains there is a strong biological propensity to regulate the amount of protein consumed (21). Even Western vegans derive ~13% of their calories from protein (22, 23).

### Individual Protein Requirements

The US adult protein RDA established by the National Academy of Medicine, (NAM) and the requirement established by the FAO and other health agencies is 0.8 g/kg bw (24–26). The RDA, which is based largely on the results of a meta-analysis of nitrogen balance studies (NBS) by Rand et al. (27), assumes an intake of good-quality protein (9). However, the NAM recognized the shortcomings of NBS and called for alternative means of assessing protein requirements (9). One such widely used alternative is the indicator amino acid oxidation method (28). utilization of this method has consistently shown protein requirements are approximately 50% greater than the RDA (29–31).

### Requirements of Plant-Based Consumers

Interest in plant-based diets has raised concern that protein nutriture may suffer because of a lower overall protein intake and lower quality of plant protein in comparison to animal protein (32–36). Therefore, it is important to determine whether the protein RDA is applicable to those consuming plant-based diets. As noted, the US RDA is based on the intake of good quality protein, although no definition of “good” was provided by the NAM (37). Several authors (36, 38–40) although not all (41), have recommended that vegans and adherents of plant-based diets consume at least 10–20% more protein than the RDA to account for the lower digestibility of plant protein (42). The Health Council of the Netherlands recommends that lacto-ovo vegetarians and vegans consume 30% more protein than non-vegetarians (43). These recommendations are prudent, especially if little of the protein consumed is comprised of high-quality protein such as soy protein. Although many individuals will likely reduce their reliance on animal products, few are likely to completely abstain from meat and fewer still from all animal products (44–46).

The potential negative impact of consuming lower quality proteins on meeting protein requirements may be partially mitigated as a result of the efficiency of protein utilization from protein complementarity. When certain lower quality proteins are combined, their combined indispensable amino acid (IAA) profile can result in a higher protein score than the score for either protein alone. The combination of cereals and beans is the prototypical example of protein complementarity in the context of plant-based diets, although many other food combinations also work (47). Cuisines around the world reflect this dietary pattern. Numerous researchers have demonstrated the value of protein combining or fortifying low-quality protein sources with complementary proteins (48–50). In many developing regions of the world, soy flour (51, 52) and even okara (53) (a byproduct of soymilk production) have been combined with traditional protein sources to produce economically affordable foods common to the local cuisine that are higher in protein content and quality.

## Soy Protein Quality

Most soy protein quality work has involved soy protein ingredients such as soy protein isolate (SPI), soy protein concentrate (SPC) and soy flour, which are comprised of ≥90%, 65–90% and 50–65% protein, respectively (54). Protein quality scores based on the protein digestibility corrected amino acid score (PDCAAS) for the soy protein ingredients range from 0.86 to 1.05 (Table 2). These scores easily exceed the threshold of ≥0.8 established by the USDA to qualify as a high-quality protein (7).

**Table 2:** Protein quality scores for soy protein derived from different soy products as determined by the PDCAAS and/or the DIAAS using different IAA scoring patterns.

**Author/Reference**	**Protein digestibility correct amino acid score**			
Hughes/ (7)	SPI, sample 1		SPI, sample 2	
	Lab. A	Lab. B	Lab. A	Lab. B
	1.02^a^	0.95^a^	1.02^a^	0.95^a^
	SPI, sample 3		SPC^a^	
	Lab. A	Lab. B	Lab. A	Lab. B
	1.02^a^	0.98^a^	1.05^a^	1.02^a^
Rutherfurd/(34)	SPI			
	Sample 1	Sample 2		
	0.979^a^	1.00^a^ (truncated)		
Mathai/(35)	SPI	Soy flour		
	0.93^a^	0.98^a^		
	0.86^c^	0.93^c^		
**Digestible indispensable amino acid score**				
Rutherfurd/(34)	SPI Sample 1	SPI Sample 2		
	0.898^b^	0.906^b^		
Mathai/(35)	SPI	Soy flour		
	84^c^	89^c^		
Reynaud/(55)	Tofu	Soymilk		
	83^c^	99^c^		
	97^d^	117^d^		
Fanelli/(56)	Impossible Burger		
	91^c^	107^d^		

*SPI, soy protein isolate; SPC, soy protein concentrate; Lab, laboratory superscripts refer to FAO scoring patterns*;

a *year 1991 (2-5 year olds)*

b *year 2007 (1-2 yo)*

C *year, 2013 (6 mo-3 y)*

d *year 2013 (older child, adolescents, adults)*.

The FAO recently recommended gradually replacing the Protein Digestibility Correct Amino Acid Score (PDCAAS) with the digestible indispensable amino acid score (DIAAS), although it will likely be many years before the DIAAS is adopted by regulatory agencies (57). The FAO developed three new scoring patterns for use with the DIAAS: birth to 6 months, 6 months to 3 years and >3 years (older child, adolescent and adult). Scores based on the DIAAS for the soy protein ingredients range from 84 to 90.6% (Table 2). Although only the younger two scoring patterns were recommended to be used for labeling purposes, the pattern for the older child, adolescent and adult applies to the majority of the population. Protein scores for both the traditional Asian soyfoods, soymilk and tofu (55), exceed the threshold (75%) established by the FAO for high-quality proteins as does the score for the Impossible Burger^TM^, which derives most of its protein from SPC (58).

## Potential Barriers To Soy Consumption

### Non-nutrients

Plant foods contain many biologically active compounds capable of exerting health effects despite not being classified as nutrients (59, 60). The non-nutrients most relevant to the evaluation of soybeans as a source of protein are the protease inhibitors (PIs) and isoflavones. PIs can interfere with protein digestion; but this effect is not straightforward (61). PIs are inactivated by heat, but the extent to which this occurs is a function of temperature, duration, particle size, and moisture conditions. Since heat also denatures protein and thus lowers quality, there is a necessary compromise between the amount used to inactivate PIs and that which does not significantly adversely affect protein quality (62, 63).

Extensive work by Shi et al. (64) showed that soaking and cooking different beans at 95°C in water (1:5 seed:water) in a beaker for 1 h resulted in 100% of the chymotrypsin inhibitor activity and 80 to 100% of the trypsin inhibitor activity being destroyed. Clearly, relatively little PI remains in properly processed pulses. Concentrated sources of soy protein such as SPI are nearly completely devoid of PI content (65, 66). Although higher amounts are found in traditional Asian soyfoods (66–68), evidence indicates these amounts do not appreciably interfere with protein digestion (55) (Table 2). Older research by Rackis et al. (69) showed that reducing the PI content by only 40 to 50% greatly increases protein digestibility.

Isoflavones are a subclass of flavonoids, a larger and more ubiquitous group of polyphenols. Among commonly consumed foods, soybeans are a uniquely rich source of isoflavones (70). In traditional Asian soyfoods, for each gram of soy protein there are approximately 3.5 mg isoflavones (71) whereas SPI and SPC contain very low levels (70)due to losses of 80 to 90% during processing (70). Consequently, health effects of isoflavones pertain mostly to whole soybeans, traditional soyfoods and soy flour.

Isoflavones are classified as phytoestrogens, although they differ significantly from estrogen both clinically and at the molecular level (72, 73). They have been posited to reduce risk of several chronic diseases (74) but are also being investigated for potential adverse effects, especially related to breast cancer, male feminization, and thyroid function. However, the American Cancer Society (75), the American Institute for Cancer Research (76), the World Cancer Research Fund International (77) and the Canadian Cancer Society (78) concluded that women with breast cancer can safely consume soyfoods. Also, the European Food Safety Authority (EFSA) (79) and the Permanent Senate Commission on Food Safety of the German Research Foundation (SKLM) (80) concluded that isoflavone supplements (soyfoods were not evaluated) do not adversely affect breast tissue. The EFSA (79) and the SKLM (SKLM) also concluded that isoflavones do not affect thyroid function, a position consistent with a recently published meta-analysis (81). The notion that soyfoods feminize men is refuted by a 2021 meta-analysis of 41 intervention studies showing neither soyfoods nor isoflavones lower circulating testosterone levels or raise estrogen levels (82) and clinical work showing they do not increase risk of gynecomastia (83, 84) or adversely affect sperm and semen parameters (85–87).

Especially important insight about potential concerns about soy came in 2017 from the US Food and Drug Administration (FDA) when it concluded that consuming 25 g/d soy protein is safe (88). This conclusion was based on comprehensive evaluation of the scientific literature and an examination of hundreds of comments submitted by the public. Although the primary focus of that review was on soy protein, nearly all safety concerns related to isoflavones. The FDA's conclusion aligns with a 2021 comprehensive technical review that evaluated 417 reports (229 observational studies, 157 clinical studies and 32 systematic reviews and meta-analyses) (89).

### Soy Protein Allergy

Regulatory agencies have recognized the need to focus allergen labeling regulations on a limited set of priority allergens. In the US, eight foods (commonly referred to as the “Big 8”) fall under the mandated labeling requirements. These eight foods (milk/dairy, eggs, fish, crustacean shellfish, tree nuts, peanuts, wheat, and soy) are thought to account for 90% of the food allergy reactions among Americans. However, the prevalence of allergy for each of these eight foods differs markedly. North American surveys show that the prevalence of soy allergy is lower than the prevalence reported for each of the other seven major allergens (90). A rough estimate is that three adults per 1,000 are allergic to soy. In comparison, the prevalence of milk/dairy allergy is 5–10x higher than soy allergy. Although soy is one of the Big 14 in Europe, the prevalence of IgE-mediated soy allergy among Europeans appears to be lower than for many commonly-consumed foods not included in the Big 14 (91, 92).

The prevalence of soy allergy among US children/adolescents is also low. Data from the National Health and Nutrition Examination Survey (years 2007–2010) show that among the Big 8 foods, the prevalence of soy allergy (0.25%) was the lowest. The prevalence of milk/dairy allergy was ~8 times greater than soy allergy (93). Furthermore, estimates based on clinical experience are that approximately 70% of children outgrow their soy allergy by age 10 (94).

## Environmental Impact of Soy Protein

Calls have been made to consume largely plant-based diets because of their lower overall environmental footprint (95, 96). As concerns about the environment increase, it is probable that interest in soy-based meat and dairy alternatives will also rise. However, there are widely differing opinions about the environmental effects of diet (97–99).

Soybeans, like all legumes, fix nitrogen due to bacterial symbionts (rhizobia) that inhabit soybean root nodules (100), reducing reliance on chemical fertilizer. An estimated half the nitrogen used for crop fertilization globally is lost into the environment, creating environmental concerns (101, 102). It is notable that the environmental impact of soybean production is often portrayed negatively due to its link with deforestation in South America (103, 104). However, production of soybeans in the US, the second leading producer of soybeans in the world (105) does not lead to deforestation (106).

In 2011, Gonzalez et al. (13) determined that of the 22 plant and animal protein sources evaluated, soybeans were the most efficiently produced and provided the most protein (g) per greenhouse gas emissions (GHGE) (kg CO_2_ equivalents). In Sweden, Carlsson-Kanyama and Gonzalez (107) determined that even when considering the environmental import costs, soybeans provided far more protein per amount of GHGE than nearly all other foods. And based on their analysis, Saarinen et al. (108) concluded that the soybean cements its position as a climate friendly and healthy food based on nutrient density and global warming potential (GWP).

Tessari et al. (109) emphasized that when evaluating the environmental impact of foods, it is important to consider nutritional value and in particular, IAA content. When this metric was used, there was little difference between animal and plant protein sources, except for soybeans, which exhibited the smallest environmental footprint. Moughan (110) also recently expressed the need to consider protein quality when evaluating the environmental impact of protein sources.

In contrast to other legumes, most soy consumed by humans is not eaten in the form of the whole bean. Therefore, it is important to consider both the cost of producing soybeans as well as the cost of producing soybean-based products. van Mierlo et al. (111) concluded soy protein is a key ingredient when trying to mimic the nutrient profile of meat while minimizing environmental impact with regard to climate change, land use, water use and fossil fuel depletion. Other investigators have also documented the environmental advantages of soy protein ingredients (112–116). For example, when evaluating 32 foods based on protein quality as determined by the DIAAS and GWP, Berardy et al. (112) found the three best performing foods were peanuts, whey, and SPI.

## Versatility of Soybeans

In 2020, of the slightly more than ~353 million MT of soybeans produced globally, approximately 113 million (31.8%) were produced in the US. Soybean production exceeds the global production of annual oilseeds (minus trees, ~272 million MT) and is much greater than global pulse production (~90 million MT) (117). Although there are concerns about the environmental impact of soybean production in Brazil (118), which is the leading soybean producer in the world, as noted previously, similar concerns are not expressed about the US production of soybeans (106).

The soybean is comprised of approximately 20% oil (by weight); approximately 85% of which is used for human consumption, although most focus is on the non-lipid component. At current production levels (for year 2018, 4.39 billion bushels, 11 pounds protein per bushel), the US soybean crop alone could deliver approximately 6 g/d protein for the entire anticipated 2050 population. Although currently just under 80% of the world's soybean crop is fed to livestock, the amount used directly for human consumption could increase in response to demand for additional dietary diversity (119). Because of the dominant use of soybeans throughout parts of Asia, soyfoods already make a substantial contribution to protein intake. For example, in Japan, soy accounts for approximately 10% of total protein intake (71, 120). Soyfood intake in China varies considerably among regions because Chinese dietary habits are quite heterogenous (121, 122). but in Shanghai, approximately 16% (123) and 13% (124) of total protein intake of men and women, respectively, is derived from soy.

However, legumes play a small role in the diets of developed countries and their direct intake is not expected to increase in the coming years in any region in the world (14, 125–127). Therefore, if soybeans are to play a larger role in meeting global protein needs, it will not likely be via the consumption of whole soybeans but via foods made from soybeans and from soy protein ingredients (concentrated source of soy protein). The vast array of traditional soyfoods that can be made from soybeans underscores the role this legume can play in meeting global protein needs. On the other hand, meat has played an important role in the diet of mankind since the beginning of time and will continue to have a strong cultural and gastronomic significance (128). Research indicates that although vegetarian and vegan consumers will accept plant-based alternatives that lack meat-like sensory properties, omnivorous and flexitarian consumers prefer alternatives that as much as possible resemble meat (129–132).

That is why it is important that the new generation of plant-based meats are formulated to emulate the taste and texture of meat (as opposed to, for example, a black bean burger) and are designed to be used in a similar manner (133). Bianchi et al. (134) recently demonstrated the potential role of meat substitutes in reducing reliance on and changing attitudes toward meat. Finally, hybrid meats, the combination of animal and plant protein, may be an especially appealing and efficacious approach to increasing dietary protein diversity because research has found that to create an effective dietary change, new practices should not diverge too much from consumers' previous behavior (135–137). If hybrid meat products increase in popularity, soy protein ingredients can contribute to their success as manufacturers have considerable experience using these combinations (119, 138).

## Conclusion

As the global population grows over the next 30 years, there will an increased need for sustainably produced food and dietary protein. The soybean, which is higher in protein than other legumes, may be in a unique position to help fill this need. It is efficiently produced and versatile as the range of foods that can be produced from this legume can fit within vastly different cuisines. Most notable in this regard are the meat substitutes made using concentrated sources of soy protein. Although other plant sources are also used for this purpose, the high quality of soy protein and the availability of soybeans make it an ideal choice. The affordability of different soyfoods varies considerably but many are less expensive than other commonly consumed sources of protein and their cost is expected to decrease as volume increases. By learning more about the health and environmental advantages of soybeans and soyfoods, nutritionists, dietitians and other health professionals will be better equipped to council their patients and clients about the benefits of incorporating soy into their diet. As consumers become increasingly interested in plant-based diets, such knowledge will become more imperative.

## Data Availability Statement

The original contributions presented in the study are included in the article/supplementary material, further inquiries can be directed to the corresponding author.

## Author Contributions

MM wrote the initial and all subsequent drafts of the manuscript and read and approved the final manuscript.

## Conflict of Interest

MM is employed by the Soy Nutrition Institute Global, an organization that receives funding from the United Soybean Board (USB) and from industry members who are involved in the manufacture and/or sale of soyfoods and/or soybean components.

## Publisher's Note

All claims expressed in this article are solely those of the authors and do not necessarily represent those of their affiliated organizations, or those of the publisher, the editors and the reviewers. Any product that may be evaluated in this article, or claim that may be made by its manufacturer, is not guaranteed or endorsed by the publisher.
